# The phenotypic spectrum of proximal 6q deletions based on a large cohort derived from social media and literature reports

**DOI:** 10.1038/s41431-018-0172-9

**Published:** 2018-06-08

**Authors:** Aafke Engwerda, Barbara Frentz, A. Lya den Ouden, Boudien C. T. Flapper, Morris A. Swertz, Erica H. Gerkes, Mirjam Plantinga, Trijnie Dijkhuizen, Conny M. A. van Ravenswaaij-Arts

**Affiliations:** 1 Department of Genetics, University of Groningen, University Medical Centre Groningen, Groningen, The Netherlands; 2 Vanboeijen, Assen, The Netherlands; 3 Department of Paediatrics, University of Groningen, University Medical Centre Groningen, Groningen, The Netherlands

## Abstract

Proximal 6q (6q11-q15) deletions are extremely rare and little is known about their phenotypic consequences. Since parents and caregivers now use social media to seek information on rare disorders, the Chromosome 6 Project has successfully collaborated with a Facebook group to collect data on individuals worldwide. Here we describe a cohort of 20 newly identified individuals and 25 literature cases with a proximal 6q deletion. Microarray results and phenotype data were reported directly by parents via a multilingual online questionnaire. This led to phenotype descriptions for five subregions of proximal 6q deletions; comparing the subgroups revealed that 6q11q14.1 deletions presented less severe clinical characteristics than 6q14.2q15 deletions. Gastroesophageal reflux, tracheo/laryngo/bronchomalacia, congenital heart defects, cerebral defects, seizures, and vision and respiratory problems were predominant in those with 6q14.2q15 deletions. Problems related to connective tissue (hypermobility, hernias and foot deformities) were predominantly seen in deletions including the *COL12A1* gene (6q13). Congenital heart defects could be linked to deletions of *MAP3K7* (6q15) or *TBX18* (6q14.3). We further discuss the role of ten genes known or assumed to be related to developmental delay and/or autism (*BAI3*, *RIMS1*, *KCNQ5*, *HTR1B*, *PHIP*, *SYNCRIP*, *HTR1E*, *ZNF292*, *AKIRIN2* and *EPHA7*). The most influential gene on the neurodevelopmental phenotype seems to be *SYNCRIP* (6q14.3), while deletions that include more than two of these genes led to more severe developmental delay. We demonstrate that approaching individuals via social media and collecting data directly from parents is a successful strategy, resulting in better information to counsel families.

## Introduction

Deletions of the proximal part of the long arm of chromosome 6, extending from 6q11 to 6q15, are rare and information on the related clinical phenotypes is scarce. The largest review to date was published by Hopkin et al. [1] in 1997; it describes 14 individuals with proximal 6q deletions, diagnosed by conventional cytogenetic methods. These individuals presented variable degrees of cognitive impairment and minor dysmorphisms: epicanthic folds, short nose with broad nasal tip, anteverted nares, long philtrum and thin upper lip. Pes planus, joint instability, hypermobility, cardiac anomalies, and umbilical and inguinal hernias were also reported. However, the deletions in that study covered different subregions of proximal 6q and they did not all overlap, therefore the shared clinical features in their cohort may not always be related to the same deleted proximal 6q segment.

Thanks to the widespread use of microarray techniques, the breakpoint estimation of deletions has become more precise and reproducible, allowing easy and reliable comparison of microarray results from all over the world. In contrast, the objective and precise gathering of phenotypic information is much more complicated. The availability of such information in international databases like ECARUCA (http://www.ecaruca.net) [2] and DECIPHER (https://decipher.sanger.ac.uk) depends on the time and willingness of doctors to submit information. As a result, only a minority of rare cases are being collected and the clinical information in these databases is often incomplete.

We have successfully used social media to gain more knowledge on the 6q11-q15 deletion phenotypes. Patients and parents of children with rare disorders increasingly seek information online and share experiences via patient support groups on social media [3]. Facebook, the largest social network in the world, has a chromosome 6 patient support group, with which we started to collaborate in 2013. Via this group, we were able to collect detailed phenotypic information directly from parents of individuals with a proximal 6q deletion. Parents and patients were invited to join the Chromosome 6 Project via Facebook and Twitter. The project enabled parents to submit detailed information on their child via an online interactive questionnaire, which was made available in seven different languages. This allowed us to perform genotype–phenotype studies on 20 newly identified individuals, in combination with 25 cases described in the literature. Clinical information and a microarray result were available for all 45 cases. Our approach led to a detailed description of the phenotypic effects of proximal 6q deletions and to the identification or confirmation of several candidate genes for specific clinical features.

## Methods

Our new cohort was recruited through social media. Since parents provided the clinical information, we have called this our parent cohort. A second group of patients was extracted from the literature (literature cohort).

### Parent cohort

Individuals were informed about the project via Facebook (Chromosome 6 Facebook group), Twitter (@C6study) and our website (https://www.chromosome6.org). Patients or their legal representatives could participate in our research by signing up via our secure website. Inclusion criteria were an isolated chromosome 6 aberration and the availability of a microarray report. Participants received a personal account for the online Chromosome 6 Questionnaire and we obtained their informed consent before they started filling it in. The accredited Medical Ethics Review Committee of the University Medical Centre Groningen waived full ethical evaluation because, according to Dutch guidelines, no ethical approval is necessary if medical information that was already available is used anonymously and no extra tests have to be performed.

Microarray reports were uploaded within the secure environment as part of the sign-up procedure. Genotype data was stored in the Chromosome 6 database and double-checked, after which the original reports were destroyed. The microarray analyses were performed in diagnostic laboratories, using different platforms. The microarray results were converted to GRCh37/hg19 with the UCSC LiftOver Tool and visualised using the UCSC genome browser (http://genome.ucsc.edu). For the present study, we selected participants with a deletion starting in the region 6q11 to 6q15 and not extending beyond 6q16.

Phenotype information was collected via the Chromosome 6 Questionnaire, which was available in English, Dutch, German, French, Italian, Spanish and Portuguese, and constructed with the MOLGENIS toolkit [4]. The questionnaire can only be accessed via a personal account and all answers are automatically stored in a secure environment. The questionnaire contains 132 closed main questions on the pregnancy, birth, congenital abnormalities, relevant dysmorphic features, development, behaviour and health of the child. Questions are only shown when applicable to the participant, based on their age, gender and previously answered questions.

Clinical photographs were collected once written consent was given and were stored on a different server to the database.

Data collected from individuals in the parent cohort was submitted to the ECARUCA database (http://www.ecaruca.net) [2] Id’s 5307, 5308 and 5310–5327.

### Literature cohort

Case reports involving proximal 6q deletions were collected using PubMed and the following search criteria: (deletion or monosomy) and (6q11 or 6q12 or 6q13 or 6q14 or 6q15 or proximal 6q). Only publications reporting microarray results or comparable detailed breakpoint analysis were included. Clinical information was extracted from the reports using the online Chromosome 6 Questionnaire in order to have as much identical information in the same database as possible.

### Data analysis

All clinical features and behavioural characteristics were classified as present, absent or unknown, and presented as present/known. The developmental delay (intelligent quotient (IQ)) was categorised as normal (>85), borderline (70–85), mild (50–70), moderate (30–50) or severe (<30) delay. This was based on formal IQ tests or, if these were not available, the mean of the developmental questions for the milestones ‘walking independently’ and ‘using two-word sentences’. The developmental quotients were calculated as the 90th centile of population age of achievement for that milestone divided by the age of achievement in the participant, times 100.

**Fig. 1:**
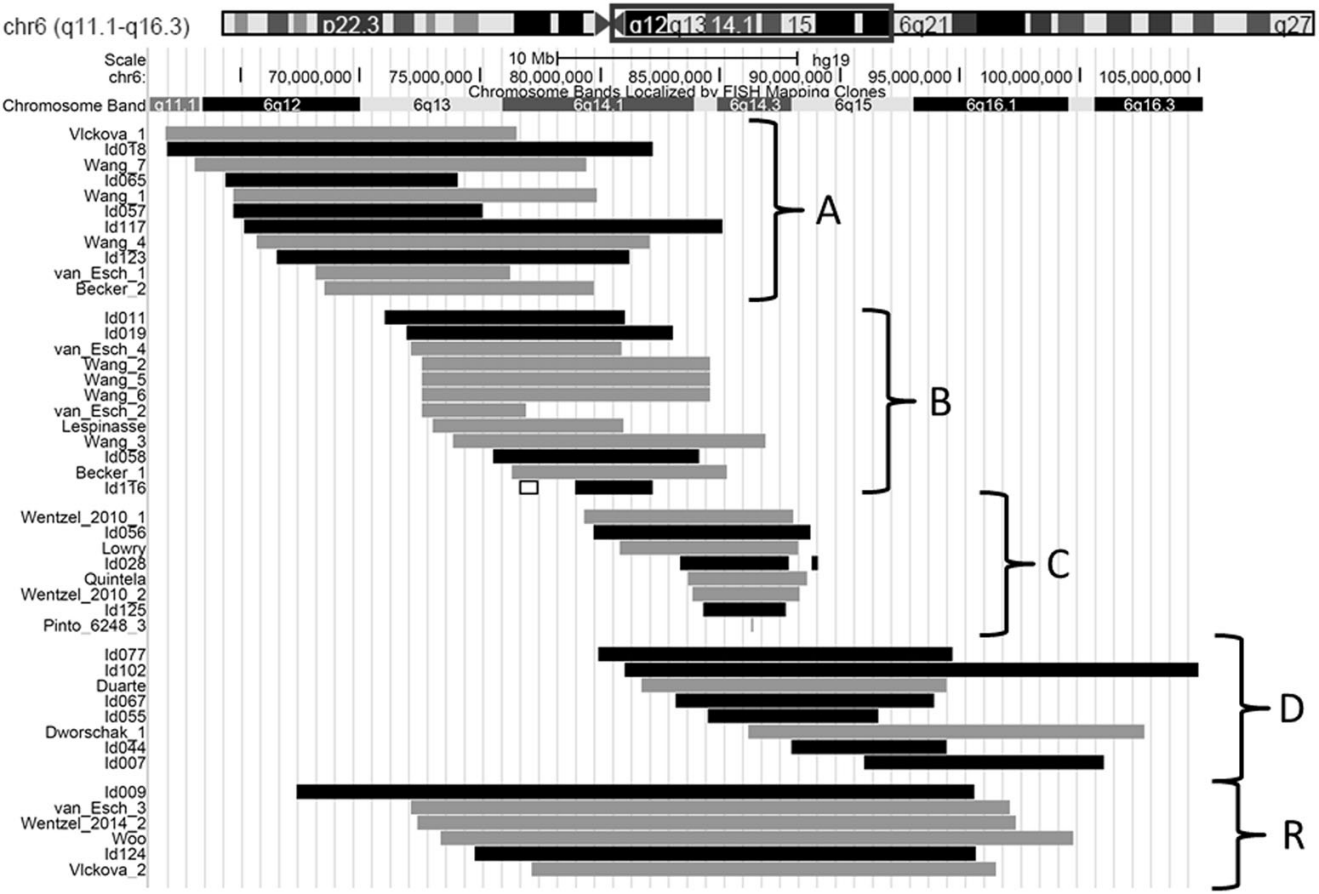
Overview of all proximal 6q deletions. Deletions in the region of 6q11-q15 are shown for our parent cohort (black bar) and literature cohort (grey bar), and are divided into five subgroups (A–R). A: deletions of 6q11q13 extending proximally to 72.5 Mb (calculated from 6pter); B: deletions of 6q13q14.1 in the region 72.5–84.5 Mb; C: deletions of 6q14.2q14.3 in the region 81–88.5 Mb; and D: larger deletions of 6q14.2q15 extending from 81–95 Mb. Deletions in subgroup (R) were large deletions of 6q12q15 overlapping with two or more of the other subgroups A–D. The deletions are visualised using the UCSC genome browser (https://genome.ucsc.edu). Patient Id116 also has a partial duplication of 6q14.1 (indicated by a white bar). The literature cases were derived from 13 reports [7–19]. See supplementary Table S1 for details

Clinical features were described for the whole group as well as for subgroups, as depicted in Fig. 1—A: deletions of 6q11q13 extending proximally to 72.5 Mb (calculated from 6pter) (*n* = 11); (B) deletions of 6q13q14.1 in the region 72.5–84.5 Mb (*n* = 12); (C) deletions of 6q14.2q14.3 in the region 81–88.5 Mb (*n* = 8); and (D) larger deletions of 6q14.2q15 extending from 81–95 Mb (*n* = 8). The remaining deletions, subgroup R, were large deletions of 6q12q15 overlapping with two or more of the other subgroups A–D (*n* = 6). The gene content for each subgroup was studied taking into account the haploinsufficiency (HI) and loss-of-function intolerance (pLI) scores. The HI score is defined as the predicted probability that a gene is more likely to exhibit HI (0–10%), or more likely not to exhibit HI (90–100%) based on differences in characteristics between known haploinsufficient and haplosufficient genes (https://decipher.sanger.ac.uk) [5]. The pLI score represents the probability that a gene is extremely intolerant of loss-of-function variation (pLI ≥0.9). Genes with scores ≤0.1 are loss-of-function-tolerant. This score is based on protein-truncating variants in the ExAC database (http://exac.broadinstitute.org) [6]. We also investigated whether a smallest region of deletion overlap (SRO) could be defined for specific clinical features.

## Results

### Participant characteristics and genotypes

The online questionnaire was made available in March 2016 and since then 125 parents have registered for an account and uploaded the microarray results of their child. Up to September 2017, 45 parents had completed and submitted the questionnaire. Of these, 20 individuals (13 males and 7 females) had a proximal 6q deletion and could be included in our parent cohort. We further compiled a cohort of 25 literature cases (12 males and 13 females) from 13 published papers [7–19]. The median age (years; months) of individuals in the parent cohort was 5; 5 (range 0; 1–24; 11), and in the literature cohort was 5; 0 (range 0; 2–22; 0).

In all individuals the breakpoints were defined by microarray except for four literature cases [11] in whom the breakpoints were accurately defined by dual-colour FISH analysis using bacterial artificial chromosome clones. Although we excluded individuals with a concomitant aberration of another chromosome, we tolerated some small additional rearrangements based on their size and gene content (see supplementary Table S1 for details and motivation). All individuals were assigned to one of the five subgroups. Figure 1 visualises the deletions, showing the overlap between the different subgroups. The main genotype characteristics of the subgroups are summarised in Table 1 (see supplementary Table S1 for details).

**Table 1:** Genotype characteristics of the deletion 6q11-q15 subgroups

Subgroup	Cohort parent/literature	Age median (range) years; months	Size median (range) Mb	No. of OMIM genes median (range)	No. of OMIM disease genes median (range)
A: q11q13 (*n* = 11)	5/6	3; 6 (0; 1–20; 0)	14.71 (8.11–20.19)	31 (12–49)	11 (5–17)
B: q13q14.1 (*n* = 12)	4/8	5; 9 (0; 9–10; 0)	9.48 (3.26–13.01)	31 (9–47)	11 (3–16)
C: q14.2q14.3 (*n* = 8)	3/5	10; 6 (2; 11–19; 0)	4.73 (0.02–9.05)	23 (2–36)	8 (1–11)
D: q14.2q15 (*n* = 8)	6/2	6; 7 (0; 3–24; 11)	11.73 (6.43–23.88)	40 (19–61)	9 (3–14)
R: q12q15 (*n* = 6)	2/4	1; 2 (0; 2–6; 6)	24.95 (19.35–28.27)	76 (54–80)	20 (12–22)
Total: q11q15	20/25	5; 5 (0; 1–24; 11)	11.19 (0.02–28.27)	32 (2–80)	11 (1–22)

Genotype characteristics for subgroups A, B, C, D and R, as represented in Fig. 1. The number (median and range) of all OMIM genes and the OMIM disease-related genes within the deletions are given (https://www.omim.org/)

### Phenotypes

**Fig. 2:**
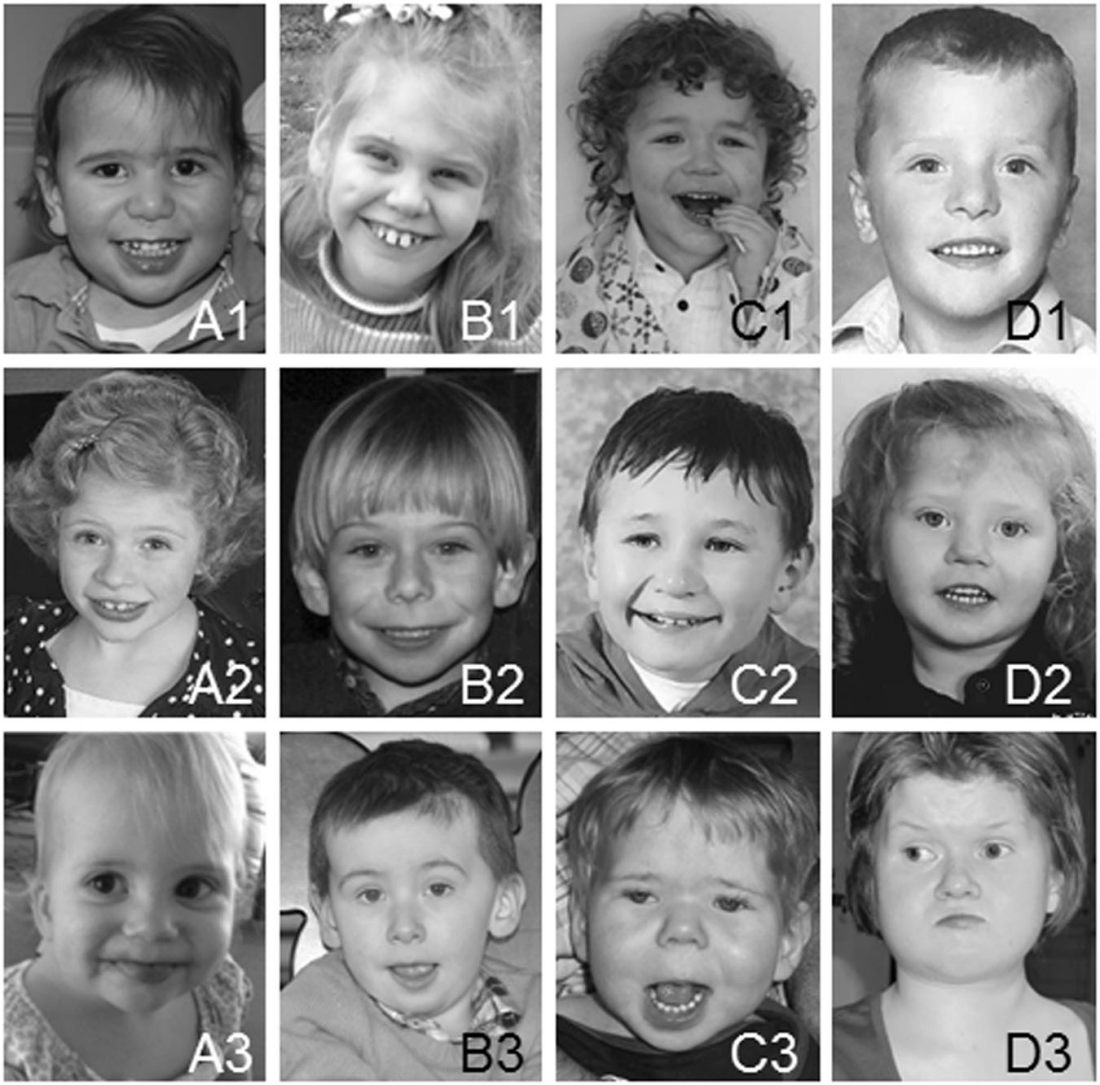
Clinical photographs of individuals with a proximal 6q deletion. Photographs of patients in subgroups A–D. Subgroup A: patient Id123 (A1) at age 22 months, patient Id065 (A2) at age 7 years, patient Id057 (A3) at age 18 months. Subgroup B: patient Id058 (B1) at age 8 years, patient Id019 (B2) at age 7 years, patient Id011 (B3) at age 5 years. Subgroup C: patient Id125 (C1) at age 2; 11 years, patient Id028 (C2) at age 8 years, patient Id056 (C3) at age 2; 5 years. Subgroup D: patient Id007 (D1) at age 8 years, patient Id044 (D2) at age 3 years, patient Id067 (D3) at age 22 years. Written consent was given to the authors to publish the patients’ photos

**Fig. 3:**
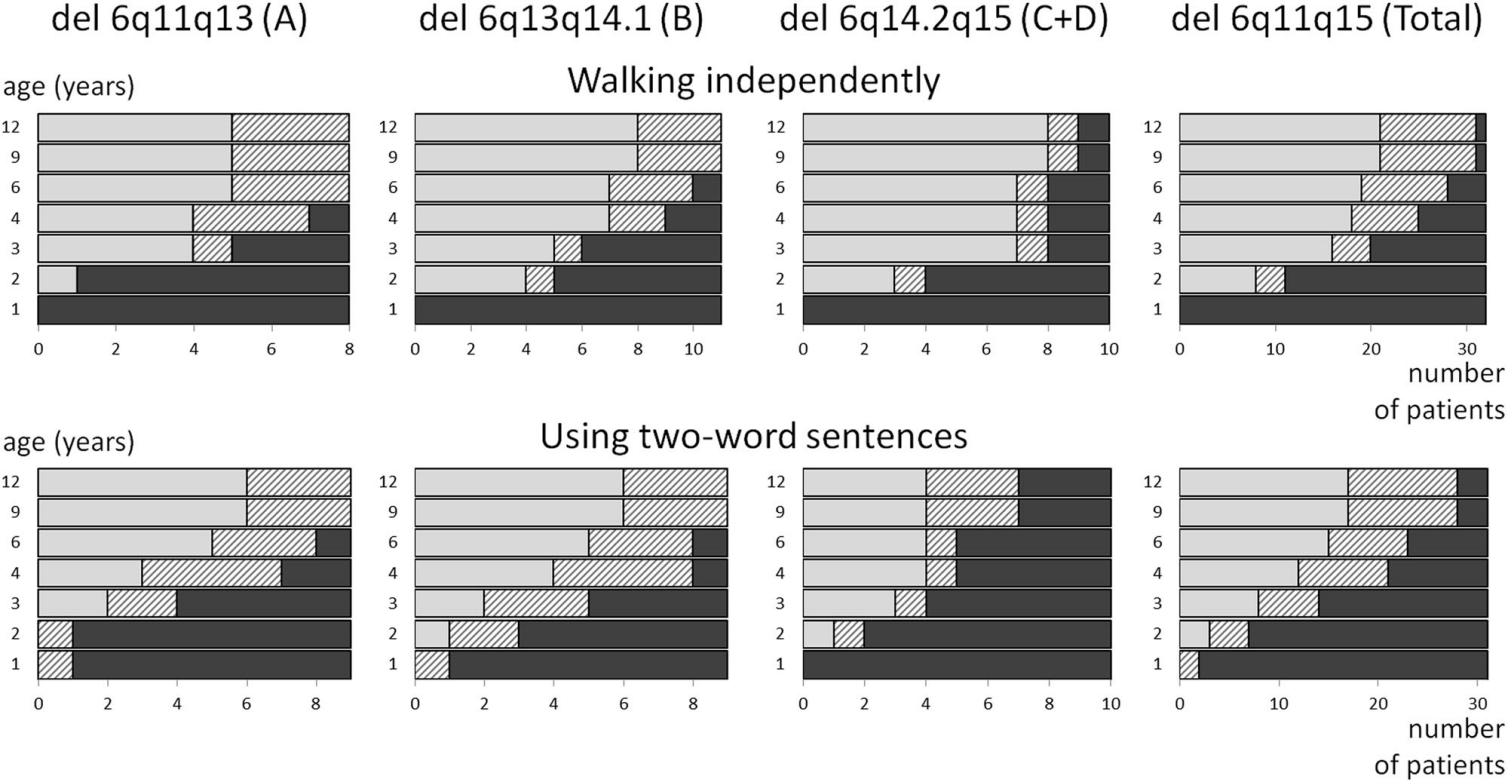
Age of achievement for the milestones ‘walking independently’ and ‘using two-word sentences’. The light grey bars indicate the number of children (*x* axis) that have reached the milestones ‘walking independently’ (upper panel) and ‘using two-word sentences’ (lower panel) before the given age (*y* axis). The dark grey bars are the children who were not able to perform the milestones at that age. The hatched bars are children who were not able to perform the milestone, but who have not yet reached the age on the *y* axis. For example, at age 12 years, 66–97% of the children are able to walk. Only children older than 12 months and for whom information is available were included here.

Phenotype information is summarised in Table 2 (see supplementary Table S2 for details) and clinical photographs are shown in Fig. 2. Developmental information is given in Table 3 and visualised in Fig. 3 and S1. Common dysmorphic and congenital abnormalities were large size—(36%) and/or abnormal shape (44%) of the head, brain abnormalities (50%), dysplastic ears (60%), cardiac defects (39%), kidney abnormalities (47%), abdominal wall hernias (52%), abnormal genitals in boys (67%), vertebral column abnormalities (41%) and joint hyperlaxity (53%). Clinical complications that were most often reported were feeding problems (88%) and gastroesophageal reflux (55%), dental problems (50%), vision problems (62%), recurrent infections (70%) and hypotonia (76%). The majority of the individuals was described as being social (60%) with behavioural problems (68%) most often reported within the autism spectrum. Developmental delay was seen in all but two participants, child Id057 (subgroup A) and child Id019 (subgroup B), (Fig. S1), who had developed normally when seen at the age of 5 and 3; 8 years, respectively.

**Table 2:** Overview of most prominent characteristics seen in individuals with proximal 6q deletions

Characteristics	Total (*n* = 45)	A (*n* = 11)	B (*n* = 12)	C (*n* = 8)	D (*n* = 8)	R (*n* = 6)
Sex (male/female)	25/20	5/6	8/4	7/1	3/5	2/4
Complicated delivery	18/30	3/7	5/7	6/7	3/3	1/6
Birth weight (<p10)	9/39	0/11	2/8	2/6	2/7	3/6
Short stature (<p10)	10/36	1/9	3/8	4/8	1/6	1/5
Small head circumference (<p10)	3/44	3/11	0/12	0/8	0/8	0/5
Large head circumference (>p90)	16/44	2/11	3/12	5/8	3/8	3/5
Abnormal skull shape	11/25	2/6	4/7	2/4	1/5	2/3
Brain abnormalities on MRI or CT	15/30	2/6	1/6	4/5	3/7	5/6
Ventriculomegaly/hydrocephaly	8/30	1/6	1/6	4/5	1/7	2/6
Vision problems	16/26	2/5	1/6	6/6	4/6	3/3
Nystagmus	7/24	0/5	1/5	2/4	1/7	3/3
Coloboma	2/24	0/5	0/5	0/5	1/6	1/3
Cataract	3/24	0/5	0/5	2/5	0/6	1/3
Dysplastic outer ear	18/30	4/8	6/8	3/4	3/7	2/3
Mild-moderate hearing impairment	6/25	1/5	2/5	1/6	0/6	2/3
Dental problems	10/20	3/5	1/4	3/4	1/5	2/2
Feeding difficulties	23/26	6/7	3/4	5/6	5/5	4/4
Requiring tube feeding	12/26	4/7	1/4	1/6	3/5	3/4
Gastroesophageal reflux	11/20	1/5	1/4	3/3	3/5	3/3
Constipation	9/20	2/5	2/4	2/3	2/5	1/3
Congenital heart defect	11/28	0/5	1/5	4/6	3/8	3/4
Atrial septal defect	5/28	0/5	0/5	1/6	2/8	2/4
Tracheo/laryngo/bronchomalacia	6/23	1/5	0/4	0/3	3/7	2/4
Recurrent infections	16/23	3/5	2/4	3/4	4/6	4/4
Kidney abnormality	15/32	7/10	2/7	1/4	3/7	2/4
Abnormal genitals in boys	12/18	3/4	4/5	3/4	0/3	2/2
Inguinal or umbilical hernia	17/33	4/8	8/10	2/5	1/6	2/4
Vertebral column abnormalities	11/27	4/7	1/5	4/6	0/5	2/4
Triphalangeal thumb	2/37	1/10	0/9	0/7	0/6	1/5
Pes planus	6/34	3/8	1/10	1/7	1/6	0/3
Positional foot deformity	7/34	2/8	4/10	0/7	1/6	0/3
Hypermobility of the joints	18/34	6/10	5/10	0/3	1/5	6/6
Hypotonia	28/37	7/10	8/10	5/7	5/7	3/3
Hypertonia/spasticity	5/24	1/7	0/4	1/4	1/6	2/3
Torticollis	5/22	2/6	1/4	1/4	0/6	1/2
Seizures/epilepsy	8/27	0/7	2/8	1/4	3/6	2/2
Developmental delay (Table 3)	38/40	9/10	11/12	8/8	7/7	3/3
Social behaviour	15/25	5/8	5/7	3/4	0/4	2/2
Behavioural problems	19/28	3/8	3/5	7/8	5/5	1/2
Autism spectrum disorder	13/28	3/8	1/5	4/8	5/5	0/2
Hyperactivity	7/28	0/8	1/5	3/8	2/5	1/2
Self-harming	6/28	0/8	1/5	4/8	1/5	0/2

Clinical features in this table were selected based on their clinical significance and prevalence. For a more detailed overview, see Supplementary table S2

**Table 3:** Development for different subgroups of proximal 6q deletions

	A q11q13	B q13q14.1	C + D q14.2q15	R q12q15	Total q11q15
Normal	1	1	0	0	2
Borderline	0	2	1	0	3
Mild delay	**6**	1	2	**1**	**10**
Moderate delay	1	**4**	1	**1**	7
Severe delay	1	1	**5**	0	7
Delayed but not specified	1	3	5	1	10
Unknown due to young age	1	0	2	3	6

Development categorised as normal (IQ >85), borderline (IQ 70–85), mild (IQ 50–70), moderate (IQ 30–50) or severe (IQ <30) delay. The category with most individuals is highlighted in bold

Not specified = developmental delay is reported, but lacking sufficient information to classify reliably. See also supplementary Fig. S1

Subgroup A (6q11q13) was mostly characterised by the following dysmorphisms and congenital malformations: dysplastic ears (4/8), kidney abnormalities (7/10), abnormal genitals in boys (3/4), umbilical hernia/omphalocele (4/8), syndactyly of the toes (5/8) and hypermobility of the joints (6/10). Clinical problems that were most frequently encountered were dental problems (3/5), feeding problems (6/7) often requiring tube feeding (4/7), recurrent infections (3/5), hypotonia (7/10) and postural problems of the vertebral column (4/7). Developmental delay was mostly mild and most children were described as being social (5/8).

In subgroup B (6q13q14.1), we observed dysplastic outer ears (6/8), abnormal genitals in boys (4/5), umbilical hernias (7/10), positional foot deformity (5/10) and hypermobility of the joints (5/10). Clinical problems that were most frequently reported were: feeding problems (3/4), short stature (3/8) and hypotonia (8/10). Developmental delay was mostly borderline to moderate. Most children were described as social (5/7), however, some behavioural problems were also reported (3/5).

A distinction was made between subgroups C (q14.2q14.3) and D (q14.2q15) based on the size of the deletion (Fig. 1). Individuals in subgroups C and D had deletions that overlapped. Remarkably, the individuals in subgroup C were identically effected for most features compared to those in subgroup D, indicating that the region q14.2q14.3 had the strongest influence on the phenotype. This is exemplified for developmental delay in supplementary Fig. S1, showing that moderate to severe developmental delay is seen in the majority of individuals over age 2 years with a deletion in this region. Subgroups C and D together (6q14.2q15) were mostly characterised by macrocephaly (8/16), brain abnormalities (7/12, especially ventriculomegaly/hydrocephalus and corpus callosum abnormalities), dysplastic outer ears (6/11), congenital heart defects (7/14), kidney abnormalities (4/11) and abnormal genitals in boys (3/7). Medical problems seen in both groups were: vision problems (10/12), gastroesophageal reflux (6/8), constipation (4/8), recurrent infections (7/10), hypotonia (10/14) and seizures (5/10). Feeding difficulties (10/11) were frequently seen, but tube feeding was required more often in subgroup D. Epilepsy (3/6) and tracheo/laryngo/bronchomalacia (3/7) was only seen in subgroup D. Most children were described as being social, but behavioural problems were common (6/7), especially autism spectrum disorder, hyperactivity and self-harming behaviour.

As expected, individuals in subgroup R with larger deletions overlapping the smaller subgroups A–D were more severely affected. The most commonly reported congenital abnormalities were brain abnormalities and congenital heart defects. Medical problems were low birth weight, feeding difficulties, nystagmus, hearing impairment, recurrent infections, hypotonia, hypermobility and epilepsy. The severity of developmental delay was only known for two individuals, while behaviour was described as social and hyperactive.

### Smallest regions of overlap

**Fig. 4:**
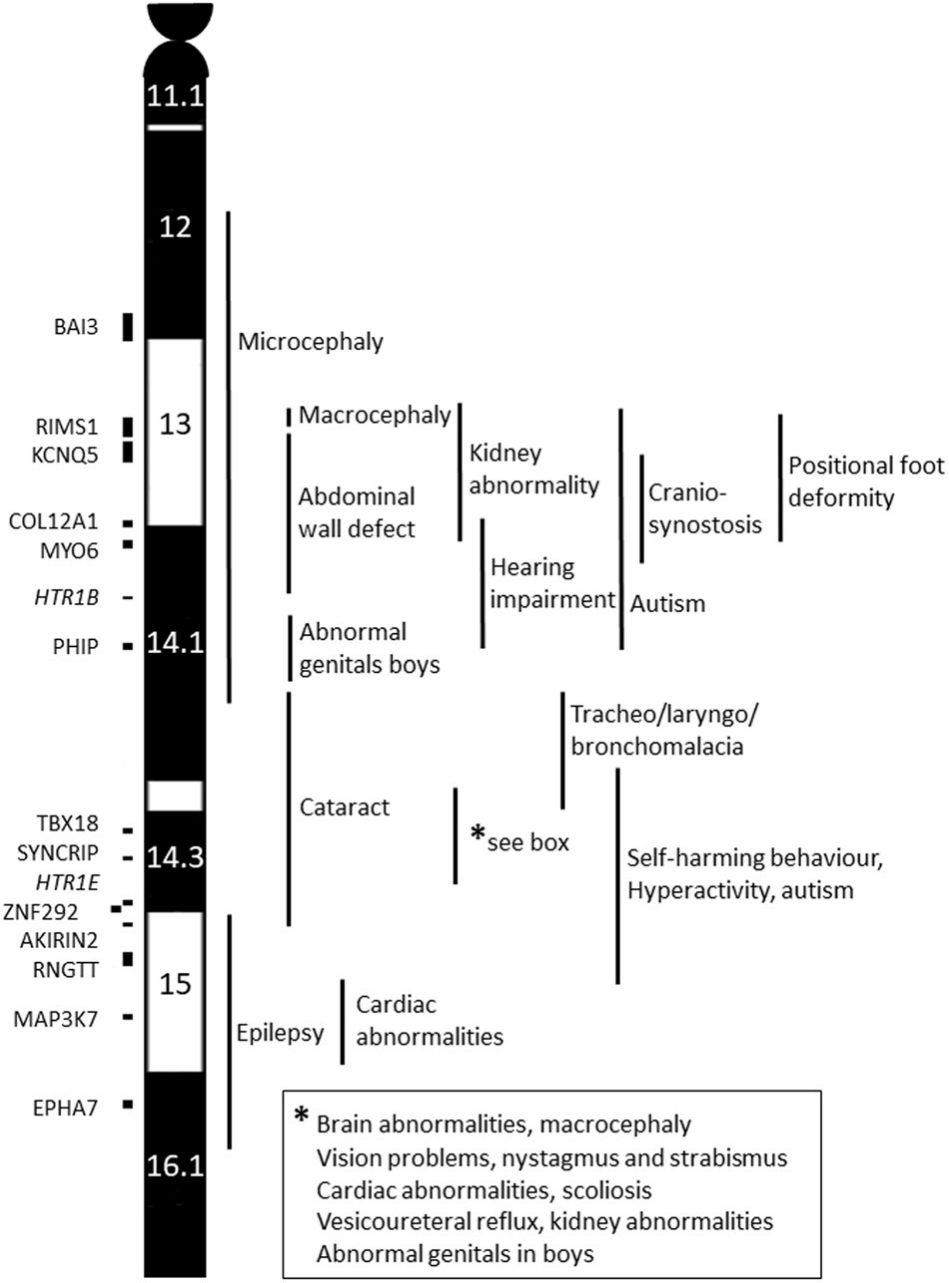
Phenotype–genotype map of proximal chromosome 6q. An idiogram of proximal 6q is shown: genes with a known or likely phenotypic effect and an HI score <10% or a pLI score >0.9 are shown on the left. Genes printed in italics have a higher HI% or lower pLI, but are discussed in the text as being related to autism. On the right-hand side, the critical regions for various clinical features are indicated

We investigated whether SROs could be defined for the most frequent clinical characteristics listed in Table 2 (data not shown). This was used to construct the phenotype–genotype map (Fig. 4) and to discuss the candidate genes involved (Discussion). Examples are given in supplementary Figs. S2 and S3 for connective tissue-related problems and congenital heart defects, respectively.

Positional foot deformity was present in seven individuals, of whom six had overlapping deletions with an SRO of 4.27 Mb (72,597–76,869 kb) that includes the candidate gene *COL12A1* (collagen type XII alpha 1 chain, MIM*120320). Other problems frequently seen in individuals with this gene deletion were hernias of the abdominal wall, hyperlaxity and scoliosis (Fig. S2).

Congenital heart defects were seen in 11 patients, of whom six had a deletion of the known cardiac-related gene *MAP3K7* (mitogen-activated protein kinase 7, MIM*602614). Four other patients had an overlapping deletion with an SRO of 3.41 Mb (location 84,326–87,736 kb) that includes the candidate gene *TBX18* (T-box 18, MIM*604613) (Fig. S3). The 11th patient had a deletion breakpoint that was 194 kb proximal of *TBX18*.

For the multi-factorial features of developmental delay and autism spectrum disorder, it was difficult to define precisely which part of proximal 6q contributed most to the phenotype. We therefore depicted the deletions by category (normal/borderline/mild/moderate/severe for developmental delay and present/absent for autism) together with the genes known or discussed to be related to these features (supplementary Figs. S1 and S4). As shown in Table 3, developmental delay was mostly mild to moderate in subgroups A and B, and moderate to severe in subgroup C + D. The milestones ‘walking independently’ and ‘using two-word sentences’ are visualised in Fig. 3. The severity of developmental delay could not be related to a more specific location or to the length of the deletion (supplementary Fig. S1).

## Discussion

The Chromosome 6 Project aims to achieve a better understanding of the clinical effect of the many different chromosome 6 abnormalities. Here we report our findings for the rare 6q deletions in the region 6q11 to 6q15. The phenotypic effect of a deletion is not the simple addition of the HI effects of the genes within the deleted segment. A deletion of multiple genes may result in a more complex mitigating or reinforcing effect of the under-represented proteins. Moreover, deletion of non-coding stretches of DNA may also affect the phenotype. Thus, it seems best to learn about the clinical effect of deletions by collecting information on individuals with such deletions. Since genetic phenotypes show much inter-individual variation, even when exactly the same gene or chromosome segment is involved, we need information on as many individuals as possible to gain a reliable picture of the effects of a specific chromosome aberration.

Notwithstanding the limitations mentioned above, knowing the gene content of a deletion, especially of genes with a known HI effect, may help to better understand the phenotype. Certain genes may even have direct clinical implications, for example, the deletion of a tumour-suppressor gene leading to an increased risk for cancer and requiring tumour screening. Other genes may be associated with an increased risk for congenital malformations and may thus direct clinical management when known to be deleted. Most genes, however, do not have a known HI effect, or only when they are affected on both alleles, and many small deletions are only considered ‘risk factors’, ie, are associated with an increased risk for learning problems, intellectual disability or behavioural problems, thereby stressing the multi-factorial nature of the phenotype.

In this discussion, we will relate the clinical phenotypes seen in our cohort to what is already known about the genes involved in the deletions. An overview of the genes within the 6q11-6q15 region and with a HI score HI <50% (https://decipher.sanger.ac.uk) is given in Table S3, together with their loss-of-function intolerance score pLI (http://exac.broadinstitute.org).

### Analysis of deletion subgroups

We collected detailed information on 20 newly identified individuals, who were approached via social media, and studied 25 literature cases. The subgroups showed considerable genetic overlap (especially subgroups A and B), while subgroup C deletions are smaller but almost fully overlap with subgroup D deletions (Fig. 1). The more proximal subgroups A and B presented fewer and less severe clinical characteristics than the more distal subgroups C and D. The individuals in subgroup R, with the larger deletions, were most severely affected (Table 2). The clinical characteristics of high medical concern, such as gastroesophageal reflux, tracheo/laryngo/bronchomalacia, congenital heart defects, cerebral defects, seizures, and vision and respiratory problems were predominant in the more distal subgroups, C and D. However, renal problems—mostly hydronephrosis—occurred more often in subgroup A (7/10 individuals), while hypermobility was seen in 6/10 and 5/10 individuals of subgroups A and B, respectively. Behavioural characteristics, such as autistic behaviour, hyperactivity and self-harming were more often seen in subgroups C and D (12/13) than in subgroups A and B (6/13). Most literature reports on deletions of 6q14q15 have focused on early-onset obesity, which was not a common problem in our cohort. Cleft palate [10] and syndactyly of the toes [19] have been assigned to 6q15, but were only present in one and two individuals of our cohort, respectively.

The 6q11-q15 region contains 24 genes with a likely clinical effect of HI based on an HI >10% or a pLI >0.9 (supplementary Table S3). Eleven of these likely HI genes have not been associated with a disorder in humans so far: *PTP4A1* (MIM*601585), *PHF3* (MIM*607789), *SMAP1* (MIM*611372), *EEF1A1* (MIM*130590), *SENP6* (MIM*605003), *IBTK* (MIM*606457), *DOPEY1* (MIM*616823), *SNAP91* (MIM*607923), *CNR1* (MIM*114610), *MDN1* (no MIM* available) and *BACH2* (MIM*605394). The other genes are discussed below.

### Developmental delay

There are six genes located in 6q11-q15 that have been associated with developmental delay (supplementary Fig. S1). *BAI3* (adhesion G protein-coupled receptor B3, MIM*602684) was deleted in 12 individuals (11 in subgroup A, 1 in subgroup R) and has been described as a candidate gene for developmental delay by Vlckova et al. [10]. Remarkably, in our cohort, developmental delay varied from none to severe, when this gene was deleted. Loss-of-function variants of *KCNQ5* (potassium voltage-gated channel subfamily Q member 5, MIM*607357) have been linked to autosomal dominant mental retardation type 46 [20]. This gene was deleted in 24 individuals with no to severe developmental delay (11 in subgroup A, 9 in subgroup B, 4 in subgroup R). Three individuals have been described in the literature with most likely loss-of-function variants in *PHIP* (pleckstrin homology domain interacting protein, MIM*612870) and a comparable phenotype consisting of developmental delay, obesity and dysmorphic features [21, 22]. In our full cohort, 25/45 patients (6 in subgroup A, 11 in subgroup B, 2 in subgroup C and 6 in subgroup R) had a *PHIP* deletion, displayed no to severe developmental delay, and had the following features in common with the three patients with loss-of-function variants: dysplastic ears (10/16), hypotonia (15/19) and strabism (4/12). Loss-of-function variants in *SYNCRIP* (synaptotagmin-binding cytoplasmic RNA-interacting protein, MIM*616686) have been detected in individuals with neurodevelopmental disorders and this might explain the more severe developmental delay seen in our subgroup C [23]. *SYNCRIP* was deleted in 20 individuals who almost all had moderate to severe delay (1 in subgroup B, 7 in subgroup C, 6 in subgroup D, 6 in subgroup R). *AKIRIN2* (akirin 2, MIM*615165) has been shown to be essential for cerebral cortex development in knockout mice [24]. No phenotype is known in humans but six individuals in our cohort have a deletion of this gene and moderate to severe developmental delay. *EPHA7* (ephrin receptor A7, MIM*602190) is known to be expressed in the brain [25], but there is no firm evidence that it is related to a neurodevelopmental disorder and we do not see a more severe developmental phenotype in individuals who lack *SYNCRIP*, *AKIRIN2* and *EPHA7* (subgroups D and R) compared to those who only lack *SYNCRIP* and *AKIRIN2* (subgroup C). As shown in Fig. 4 and S1, the most important region for severe developmental delay seems to be 6q14.2q14.3 (subgroup C) and the most important genes involved are *SYNCRIP* and *AKIRIN2*. However, the other genes known to be related to developmental delay—*BAI3*, *KCNQ5*, *PHIP* and *EPHA7—*may also have an effect.

As shown in Fig. S1, deletion of only *PHIP* or only *SYNCRIP* is enough to cause severe developmental delay, but most individuals with deletions of these genes show borderline to severe, or mild to severe developmental delay, respectively. On the other hand, all those with a deletion of at least three of the six genes related to developmental delay have mild to severe delay, so there is an indirect effect from deletion size.

### Autism

Autism spectrum disorders are frequently described in chromosomal syndromes and an increasing number of genes have been associated to this behavioural phenotype. Autism-related behaviour was predominantly seen in subgroups C and D (Table 2 and supplementary Fig. S4). Genes located in proximal 6q that have been linked to autism are *RIMS1* (regulating synaptic membrane exocytosis 1, MIM*606629), *PHIP*, *SYNCRIP* and *ZNF292* (zinc finger protein 292, MIM*616213). A missense mutation in *RIMS1* has been identified in one family with cone-rod dystrophy type 7, with an age of onset from 14 to 42 years [26]. This eye disorder was not observed in our cohort. However, others have shown an increased number of truncating *RIMS1* variants in individuals with autism spectrum disorder [27, 28]. Thus, deletion of *RIMS1* might be a risk factor for autism. In our cohort, 22 individuals (11 in subgroup A, 8 in subgroup B, 3 in subgroup R) had a complete or partial deletion of *RIMS1*. Information on behaviour was available for 13 of them but only three had autism-like behaviour. *PHIP* variants have been identified in individuals with autism [29]. However, in our cohort, only 3/14 individuals with a *PHIP* deletion had an autism spectrum disorder. In the meta-analysis by Lelieveld et al. [23], *SYNCRIP* was identified as a gene for intellectual disability and Pinto et al. [18] described one patient with a small de novo deletion of only 23 kb including part of the *SYNCRIP* gene (see Pinto_6248_3 in Fig. 1 and S4). This patient had severe developmental delay and autism, indicating that autism may be part of the *SYNCRIP-*related phenotype. In total, 7/13 patients with a *SYNCRIP* deletion in our cohort had an autism spectrum disorder. There is one report of likely disruptive variants in *ZNF292* being associated with autism [29] and we observed autism in 4/10 individuals with a *ZNF292* deletion. More studies are needed to draw definite conclusions on the role of this gene in autism.

Apart from these four known genes, the genes *HTR1E* (5-hydroxytryptamine receptor 1E, MIM*182132) and *HTR1B* (5-hydroxytryptamine receptor 1B MIM*182131), which did not pass our strict HI score, may also play a role in autism-like behaviour (supplementary Table S3 and Fig. S4). These genes both code for serotonin receptors. Serotonin is a neurotransmitter that plays a role in various cognitive and behavioural functions, including feeding, sleep, pain, depression and learning [30]. A case–control study in 252 individuals with autism spectrum disorder suggested a role for *HTR1B* in the predisposition to autism, since such individuals more often had polymorphic *HTR1B* variants that were known to have a lower expression [31]. Remarkably, we observed an autism spectrum disorder in 7/13 individuals with an *HTR1E* and a *SYNCRIP* deletion, while the numbers were 3/13 for *RIMS1*, 3/12 for *HTR1B*, 3/14 for *PHIP* and 4/10 for *ZNF929* (supplementary Fig. S4).

### Connective tissue-related problems

Hypermobility, foot deformities and hernias were more often seen in subgroups A and B. *COL12A1* is thought to be responsible for these connective tissue-related problems in the most proximal 6q deletions. Other disorders linked to this gene are due to recessive or dominant-negative mutations (Ullrich congenital muscular dystrophy 2 (MIM#616470) and Bethlem myopathy 2 (MIM#616471)). If we analyse the clinical features of all the individuals in groups A, B and R with a *COL12A1* deletion (*n* = 24), we see hernias in 13/18, hyperlaxity in 15/21, foot deformities in 8/16 and kyphosis/scoliosis in 7/12, thereby supporting the role of this gene in the connective tissue phenotype. In supplementary Fig. S2, we show that 14/16 individuals who have at least two of the above features, have a deletion of *COL12A1*. Another collagen gene in this region is *COL9A1* (collagen type IX alpha 1 chain, MIM*120210), which is associated with cartilage problems. However, this gene has an HI of 24% and a pLI of 0, indicating that a HI effect is very unlikely.

### Deafness

*MYO6* (myosin VI, MIM*600970) is involved in autosomal dominant deafness type 22 (DFNA22) due to both missense and truncating mutations [19] and thus might explain the deafness seen in 5/23 individuals with a deletion including *MYO6*. However, in DFNA22, deafness is progressive with an age of onset of 20 years and onwards, while the individuals with hearing problems in our cohort range in age from 8 months to 22 years, with an unknown age of onset of their hearing loss.

### Heart defects

Congenital heart defects were present in 11 patients, of whom six had a deletion of *MAP3K7*, a gene known to be related to cardiac defects, while nine had a deletion of our candidate gene *TBX18* (supplementary Fig. S3). Together they covered the deletions of 10/11 individuals with a heart defect. The single patient who had neither a deletion of *MAP3K7* nor *TBX18* had a breakpoint 194 kb proximal to *TBX18*, so a position effect cannot be excluded. *MAP3K7* is a well-known disease gene, with missense or non-truncating mutations resulting in autosomal dominant frontometaphyseal dysplasia type 2 (MIM#617137) and cardiospondylocarpofacial (MIM#157800) syndromes, while deletions seem to have a different phenotypic effect [32]. Nonetheless, heart defects are part of the cardiospondylocarpofacial syndrome and we observed heart defects in 6/12 individuals with a *MAP3K7* deletion. *TBX18* is thought to be responsible for the development of the myocytes in the ventricular septum and the atrial and ventricular walls of the heart although cardiac disease-related variants in the human *TBX18* gene have not been reported. However, heterozygous variants within the *TBX18* gene promoter were reported in 4 out of 326 individuals with a ventricular septal defect, while no functional variants were found in a control group (*n* = 327) [33]. Most individuals with a heart defect in our cohort presented with an atrial septal defect. *TBX18* is also known to be associated to congenital kidney and urinary tract anomalies. In our total cohort (C, D, R), 19 individuals had a *TBX18* deletion, of whom 6/13 had a kidney abnormality and 9/15 had a congenital heart defect.

### Limitations to our findings

Our subgroups are very small due to the rarity of chromosome 6 aberrations. We had detailed information for 20 participants from our Chromosome 6 Project, but the data from the 25 case reports were often incomplete, lacking especially information about milestones and behaviour. For example, most of the children in our parent cohort were described as being social, whereas this information was often not available for the literature cases.

The main aim of the Chromosome 6 Project is to create helpful information for parents and doctors. Here we report the genotypes and phenotypes of 45 individuals with proximal 6q deletions, the largest cohort to date. However, larger numbers of cases are needed to make more detailed and reliable descriptions of the expected phenotypes and their variability, while follow-up data will give more insight into their development and future perspectives. The Chromosome 6 Project uses social media to collect information, not only on the phenotypes and genotypes but also on what topics are important for parents and how we can better involve them in our research. The project currently has >700 followers on Twitter and reaches >900 members of the Facebook group. Unfortunately, we do not know how many of these individuals have a chromosome 6 aberration.

So far, little attention has been given to the barriers that families encounter in participating in studies like the Chromosome 6 Project, while studies requested and initiated by parents are often clinically relevant and result in highly motivated participation. Of course, there are questions about the reliability of information retrieved directly from parents. For example, one might assume that parents tend to overestimate their child’s developmental level. Therefore, we only used the results of standardised developmental tests or well-defined milestones to categorise the individuals in subgroups of developmental delay. We also asked for formal behavioural diagnoses of autism spectrum disorder and ADHD, rather than relying on parents’ perspectives of their child’s behaviour. We are in the process of validating our online questionnaire by comparing parental data with information collected from the medical professionals. The results so far are encouraging.

## Concluding remarks

Not surprisingly, the present study showed that our questionnaire led to extra and more detailed information than that available in case reports. We demonstrate that patient/parent involvement via social media is a successful strategy and it will result in a growing wealth of information that will prove extremely important to parents of young children with a rare chromosome aberration. Our goal is to construct a detailed phenotype–genotype map for the complete chromosome 6 that can be used for counselling and the clinical management of patients in the future.

### URLs

      https://decipher.sanger.ac.uk/

http://genome.ucsc.edu/

http://exac.broadinstitute.org

https://www.chromosome6.org/

http://www.ecaruca.net

https://www.omim.org/

## Electronic supplementary material

Supplementary Figs. S1-S4

Table S1

Table S2

Table S3
