# Supplementary material for: The phenotypic spectrum of proximal 6q deletions based on a large cohort derived from social media and literature reports
Source: Eur J Hum Genet. 2018 Jun 8;26(10):1478–89. doi: 10.1038/s41431-018-0172-9 (PMC6138703; doi:10.1038/s41431-018-0172-9)
Supplement: Supplementary file 1 — Supplementary Figs. S1-S4 [file 41431_2018_172_MOESM1_ESM.docx]

**Supplementary figures**

**Figure S1. Developmental delay in children older than 2 years of age**

**
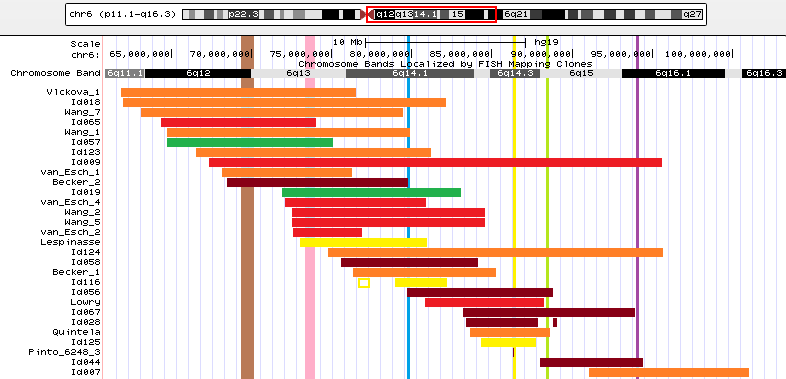
**

The deletion in each patient for whom development could be categorised is represented by a horizontal bar. Development is categorised as normal (IQ >85, green bar), borderline (IQ 70-85, yellow), mild (IQ 50-70, orange), moderate (IQ 30-50, red) or severe (IQ <30, dark red) delay.

Genes marked by a vertical line (*BAI3* (brown), *KCNQ5* (pink), *PHIP* (blue), *SYNCRIP* (yellow), *AKIRIN2* (green) and *EPHA7* (purple)) have all been related to developmental delay (see manuscript Discussion, section on Developmental delay).

**Figure S2. Deletions resulting in connective tissue-related clinical features**


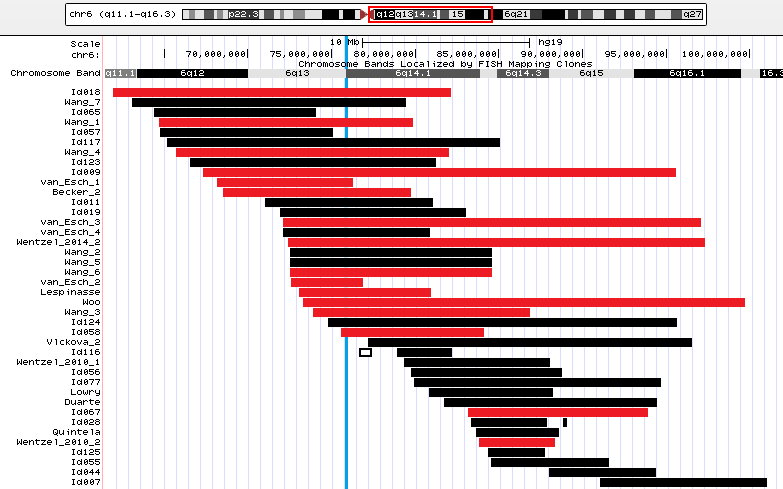


Deletions in individuals with at least two of the following features: hyperlaxity, hernia of the abdominal wall, foot deformity and kyphosis/scoliosis are depicted as a red bar. The *COL12A1* gene is indicated by the vertical blue line.

**Figure S3. Shortest region of overlap for heart defects**


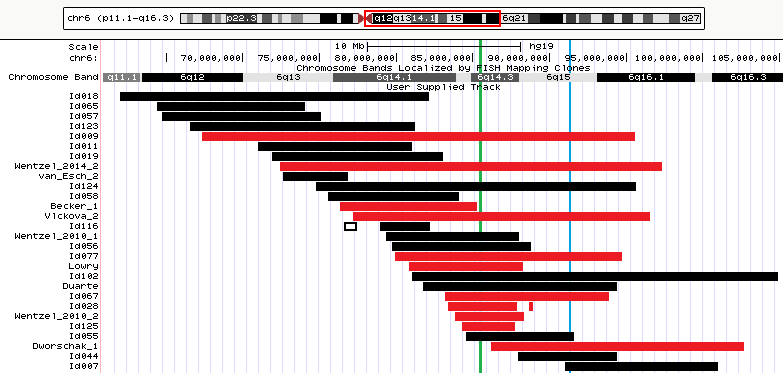


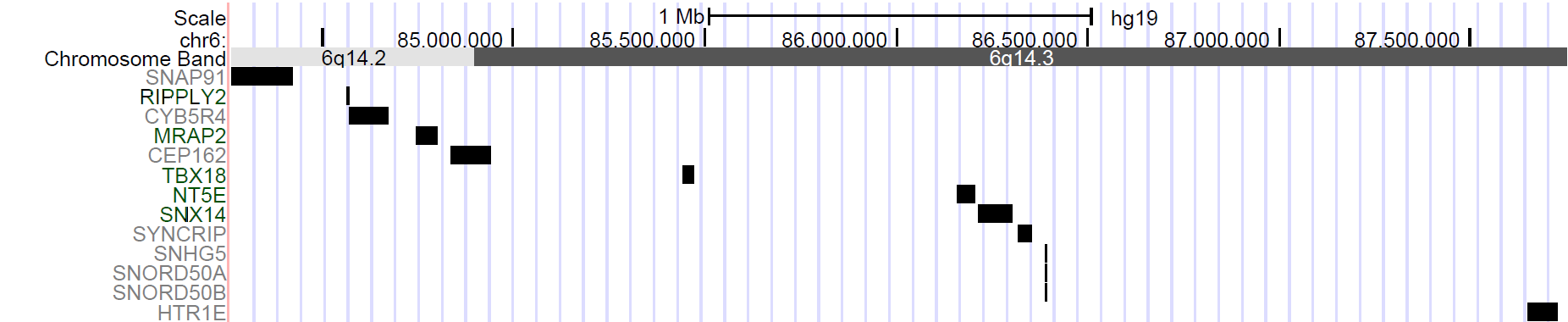


The deletions in individuals with and without a heart defect are shown as red and black horizontal bars, respectively. *MAP3K7,* the gene known to be related to heart defects is indicated by the blue vertical line. Our candidate gene *TBX18* is indicated by the green line. The shortest region of overlap (SRO) of the deletions, not including *MAP3K7,*  is marked by dashed vertical lines. Individual Becker_1 does not overlap, but *TBX18* is located only 194 kb from the distal deletion breakpoint in this individual. In the lower panel the SRO is given including *TBX18,* marked by a circle.

**Figure S4. Autism spectrum disorder in proximal 6q deletions**

**
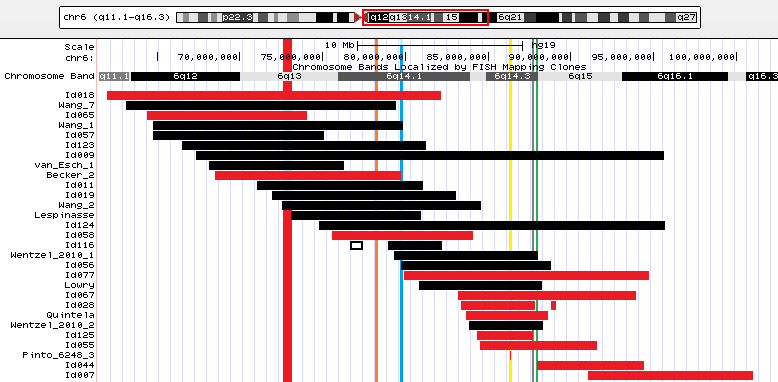
**

The deletions in individuals with and without autism spectrum disorder are shown as red and black horizontal bars, respectively. Genes marked by the vertical lines (*RIMS1* (red), *HTR1B* (orange), *PHIP* blue), *SYNCRIP* (yellow), *HTR1E* (grey) and *ZNF292* (green)) have all been related to autism (see manuscript Discussion, section on Autism).
