# Supplementary material for: The phenotypic spectrum of proximal 6q deletions based on a large cohort derived from social media and literature reports
Source: Eur J Hum Genet. 2018 Jun 8;26(10):1478–89. doi: 10.1038/s41431-018-0172-9 (PMC6138703; doi:10.1038/s41431-018-0172-9)
Supplement: Supplementary file 3 — Table S2 [file 41431_2018_172_MOESM3_ESM.docx]

**Supplementary Table 2: Overview of clinical characteristics seen in individuals with proximal 6q deletions**

| **Characteristics** | **Total (n=45)** | **A (n=11)** | **B (n=12)** | **C (n=8)** | **D (n=8)** | **R (n=6)** |
| --- | --- | --- | --- | --- | --- | --- |
| **General** |  |  |  |  |  |  |
| Sex (male/female) | 25/20 | 5/6 | 8/4 | 7/1 | 3/5 | 2/4 |
| Complicated delivery |  |  |  |  |  |  |
| Breech | 8/30 | 1/7 | 2/7 | 2/7 | 3/3 | 0/6 |
| Caesarean section/forceps/vacuum | 17/30 | 3/7 | 5/7 | 6/7 | 2/3 | 1/6 |
| Birth weight  <p10/p10-p90/>p90 | 9/21/8 | 0/8/3 | 2/1/5 | 2/4/0 | 2/5/0 | 3/3/0 |
| Height (at last examination)  <p10/p10-p90/>p90 | 10/21/5 | 1/7/1 | 3/3/2 | 4/2/2 | 1/5/0 | 1/4/0 |
| **Head** |  |  |  |  |  |  |
| Head circumference (at last examination)  <p10/p10-p90/>p90 | 3/25/16 | 3/6/2 | 0/9/3 | 0/3/5 | 0/5/3 | 0/2/3 |
| Abnormal skull shape |  |  |  |  |  |  |
| Plagiocephaly | 8/25 | 1/5 | 2/5 | 2/4 | 1/5 | 2/3 |
| Craniosynostosis | 4/25 | 1/6 | 2/6 | 0/3 | 0/4 | 1/2 |
| Brain abnormalities on MRI or CT | 15/30 | 2/6 | 1/6 | 4/5 | 3/7 | 5/6 |
| Cerebral atrophy | 4/30 | 2/6 | 0/6 | 0/5 | 0/7 | 2/6 |
| Corpus callosum abnormality | 7/30 | 0/6 | 1/6 | 2/5 | 2/7 | 2/6 |
| Delayed myelination | 5/30 | 1/6 | 0/6 | 3/5 | 0/7 | 1/6 |
| Ventriculomegaly/hydrocephaly | 8/30 | 1/6 | 1/6 | 4/5 | 1/7 | 2/6 |
| **Eyes** |  |  |  |  |  |  |
| Vision problems | 16/26 | 2/5 | 1/6 | 6/6 | 4/6 | 3/3 |
| Legally blind | 1/26 | 0/5 | 0/6 | 0/6 | 0/6 | 1/3 |
| CVI | 4/26 | 0/5 | 1/6 | 1/6 | 1/6 | 1/3 |
| Nystagmus | 7/24 | 0/5 | 1/5 | 2/4 | 1/7 | 3/3 |
| Strabism | 9/24 | 1/5 | 1/5 | 3/4 | 2/7 | 2/3 |
| Coloboma | 2/24 | 0/5 | 0/5 | 0/5 | 1/6 | 1/3 |
| Cataract | 3/24 | 0/5 | 0/5 | 2/5 | 0/6 | 1/3 |
| Hypotelorism | 3/24 | 1/9 | 2/8 | 0/2 | 0/3 | 0/2 |
| Hypertelorism | 7/30 | 3/10 | 0/9 | 1/4 | 2/4 | 1/3 |
| **Ears** |  |  |  |  |  |  |
| Dysplastic outer ear | 18/30 | 4/8 | 6/8 | 3/4 | 3/7 | 2/3 |
| Mild to moderate hearing impairment | 6/25 | 1/5 | 2/5 | 1/6 | 0/6 | 2/3 |
| Tympanic tubes (grommets) | 7/21 | 2/5 | 1/4 | 2/4 | 1/6 | ½ |
| **Mouth** |  |  |  |  |  |  |
| Cleft palate | 1/34 | 0/8 | 0/12 | 1/5 | 0/5 | 0/4 |
| Dental problems | 10/20 | 3/5 | 1/4 | 3/4 | 1/5 | 2/2 |
| **Gastrointestinal** |  |  |  |  |  |  |
| Feeding difficulties | 23/26 | 6/7 | 3/4 | 5/6 | 5/5 | 4/4 |
| Requiring tube feeding | 12/26 | 4/7 | 1/4 | 1/6 | 3/5 | 3/4 |
| Gastroesophageal reflux | 11/20 | 1/5 | 1/4 | 3/3 | 3/5 | 3/3 |
| Constipation | 9/20 | 2/5 | 2/4 | 2/3 | 2/5 | 1/3 |
| **Cardiovascular system** |  |  |  |  |  |  |
| Congenital heart defect | 11/28 | 0/5 | 1/5 | 4/6 | 3/8 | 3/4 |
| Atrial Septal Defect | 5/28 | 0/5 | 0/5 | 1/6 | 2/8 | 2/4 |
| **Respiratory system** |  |  |  |  |  |  |
| Bronchomalacia | 3/23 | 0/5 | 0/4 | 0/3 | 2/7 | 1/4 |
| Laryngomalacia | 5/23 | 1/5 | 0/4 | 0/3 | 3/7 | 1/4 |
| Tracheomalacia | 4/23 | 1/5 | 0/4 | 0/3 | 2/7 | 1/4 |
| Congenital diaphragmatic hernia | 1/21 | 0/5 | 0/4 | 0/3 | 0/6 | 1/3 |
| **Recurrent infections** | 16/23 | 3/5 | 2/4 | 3/4 | 4/6 | 4/4 |
| Otitis media | 7/23 | 1/5 | 1/4 | 2/4 | 2/6 | 1/4 |
| Recurrent lower respiratory tract | 7/23 | 1/5 | 1/4 | 1/4 | 1/6 | 3/4 |
| Recurrent upper respiratory tract | 8/23 | 3/5 | 1/4 | 0/4 | 1/6 | 3/4 |
| Urinary tract | 4/23 | 2/5 | 0/4 | 1/4 | 0/6 | 1/4 |
| **Urogenital system** |  |  |  |  |  |  |
| Kidney abnormality | 15/32 | 7/10 | 2/7 | 1/4 | 3/7 | 2/4 |
| Hydronephrosis and/or reflux | 10/32 | 4/10 | 2/7 | 0/4 | 3/7 | 1/4 |
| Ectopic kidney | 1/32 | 0/10 | 1/7 | 0/4 | 0/7 | 0/4 |
| Hypoplasia of kidney | 2/32 | 1/10 | 0/7 | 0/4 | 0/7 | 1/4 |
| Abnormal genitals boys | 12/18 | 3/4 | 4/5 | 3/4 | 0/3 | 2/2 |
| Cryptorchism | 6/18 | 2/4 | 2/5 | 0/4 | 0/0 | 2/2 |
| Micropenis | 4/18 | 1/4 | 1/5 | 1/4 | 0/0 | 1/2 |
| Hypospadia | 5/18 | 0/4 | 2/5 | 2/4 | 0/0 | 1/2 |
| **Trunk** |  |  |  |  |  |  |
| Pectus excavatum | 5/21 | 1/6 | 2/5 | 2/4 | 0/3 | 0/3 |
| Abdominal wall defect |  |  |  |  |  |  |
| Inguinal hernia | 2/33 | 0/8 | 1/10 | 1/5 | 0/6 | 0/4 |
| Omphalocele | 1/33 | 1/8 | 0/10 | 0/5 | 0/6 | 0/4 |
| Umbilical hernia | 14/33 | 3/8 | 7/10 | 1/5 | 1/6 | 2/4 |
| Vertebral column abnormalities | 11/27 | 4/7 | 1/5 | 4/6 | 0/5 | 2/4 |
| Abnormal vertebrae | 4/27 | 0/7 | 1/5 | 2/6 | 0/5 | 1/4 |
| Kyphosis | 4/27 | 1/7 | 0/5 | 1/6 | 0/5 | 2/4 |
| Scoliosis | 8/27 | 2/7 | 1/5 | 4/6 | 0/5 | 1/4 |
| Sacral dimple | 15/25 | 4/7 | 2/5 | 2/3 | 5/7 | 2/3 |
| **Extremities** |  |  |  |  |  |  |
| Hand |  |  |  |  |  |  |
| Finger syndactyly | 2/37 | 2/10 | 0/9 | 0/7 | 0/6 | 0/5 |
| Triphalangeal thumb | 2/37 | 1/10 | 0/9 | 0/7 | 0/6 | 1/5 |
| Foot |  |  |  |  |  |  |
| Pes planus | 6/34 | 3/8 | 1/10 | 1/7 | 1/6 | 0/3 |
| Positional foot deformity | 7/34 | 2/8 | 4/10 | 0/7 | 1/6 | 0/3 |
| Toe syndactyly | 9/34 | 5/8 | 1/10 | 2/7 | 0/6 | 1/3 |
| Hypermobility of the joints | 18/34 | 6/10 | 5/10 | 0/3 | 1/5 | 6/6 |
| **Skin** |  |  |  |  |  |  |
| Cafe-au-lait spot | 4/22 | 0/5 | 0/4 | 2/7 | 1/4 | 1/2 |
| Capillary skin hemangioma | 2/22 | 0/5 | 0/4 | 1/7 | 0/4 | 1/2 |
| **Neurology** |  |  |  |  |  |  |
| Hypotonia | 28/37 | 7/10 | 8/10 | 5/7 | 5/7 | 3/3 |
| Hypertonia/spasticity | 5/24 | 1/7 | 0/4 | 1/4 | 1/6 | 2/3 |
| Torticollis | 5/22 | 2/6 | 1/4 | 1/4 | 0/6 | 1/2 |
| Seizures | 8/27 | 0/7 | 2/8 | 1/4 | 3/6 | 2/2 |
| Epilepsy | 4/27 | 0/7 | 2/8 | 0/4 | 2/6 | 1/2 |
| Developmental delay (see table 3) | 38/40 | 9/10 | 11/12 | 8/8 | 7/7 | 3/3 |
| **Behaviour** |  |  |  |  |  |  |
| Social behaviour | 15/25 | 5/8 | 5/7 | 3/4 | 0/4 | 2/2 |
| Behavioural problems | 19/28 | 3/8 | 3/5 | 7/8 | 5/5 | 1/2 |
| Autism spectrum disorder | 13/28 | 3/8 | 1/5 | 4/8 | 5/5 | 0/2 |
| Hyperactivity | 7/28 | 0/8 | 1/5 | 3/8 | 2/5 | 1/2 |
| Self-harming | 6/28 | 0/8 | 1/5 | 4/8 | 1/5 | 0/2 |
