# Supplementary material for: The phenotypic spectrum of proximal 6q deletions based on a large cohort derived from social media and literature reports
Source: Eur J Hum Genet. 2018 Jun 8;26(10):1478–89. doi: 10.1038/s41431-018-0172-9 (PMC6138703; doi:10.1038/s41431-018-0172-9)
Supplement: Supplementary file 4 — Table S3 [file 41431_2018_172_MOESM4_ESM.docx]

**Table S3. Genes with haploinsufficiency score HI below 50%**

| **Cytogenetic location** | **Gene** | **Name** | **% HI*** | **pLI*** |
| --- | --- | --- | --- | --- |
| 6q11.1 | KHDRBS2 | KH RNA binding domain containing, signal transduction associated 2 | 11.26 | 0.14 |
| 6q12 | **PTP4A1** | Protein tyrosine phosphatase type IVA, member 1 | 9.60 | 0.89 |
| 6q12 | **PHF3** | PHD finger protein 3 | 36.84 | 1.00 |
| 6q12 | EYS | Eyes shut homolog (Drosophila) | 25.63 | 0.00 |
| 6q12-q13 | **BAI3** | Adhesion G protein-coupled receptor B3 | 3.02 | 1.00 |
| 6q13 | LMBRD1 | LMBR1 domain containing 1 | 12.92 | 0.00 |
| 6q13 | COL19A1 | Collagen type XIX alpha 1 chain | 26.70 | 0.00 |
| 6q13 | COL9A1 | Collagen type IX alpha 1 chain | 23.88 | 0.00 |
| 6q13 | FAM135A | Family with sequence similarity 135 member A | 26.09 | 0.79 |
| 6q13 | **SMAP1** | Small ArfGAP 1 | 34.53 | 0.98 |
| 6q13 | B3GAT2 | Beta-1,3-glucuronyltransferase 2 | 33.88 | 0.00 |
| 6q13 | **RIMS1** | Regulating synaptic membrane exocytosis 1 | 9.51 | 0.03 |
| 6q13 | **KCNQ5** | Potassium voltage-gated channel subfamily Q member 5 | 12.28 | 1.00 |
| 6q13 | MTO1 | Mitochondrial tRNA translation optimization 1 | 39.11 | 0.00 |
| 6q13 | **EEF1A1** | Eukaryotic translation elongation factor 1 alpha 1 | 12.19 | 0.98 |
| 6q13 | SLC17A5 | Solute carrier family 17 member 5 | 19.72 | 0.02 |
| 6q13-q14.1 | **COL12A1** | Collagen type XII alpha 1 chain | 16.12 | 1.00 |
| 6q14.1 | COX7A2 | Cytochrome c oxidase subunit 7A2 | 47.62 | 0.38 |
| 6q14.1 | TMEM30A | Transmembrane protein 30A | 19.68 | 0.04 |
| 6q14.1 | FILIP1 | Filamin A interacting protein 1 | 26.61 | 0.00 |
| 6q14.1 | **SENP6** | SUMO1/sentrin specific peptidase 6 | 19.18 | 1.00 |
| 6q14.1 | **MYO6** | Myosin VI | 7.35 | 0.02 |
| 6q14.1 | HTR1B | 5-hydroxytryptamine receptor 1B | 28.43 | 0.65 |
| 6q14.1 | **PHIP** | Pleckstrin homology domain interacting protein | 12.51 | 1.00 |
| 6q14.1 | HMGN3 | High mobility group nucleosomal binding domain 3 | 24.65 | 0.18 |
| 6q14.1 | LCA5 | Lebercilin | 45.14 | 0.00 |
| 6q14.1 | SH3BGRL2 | SH3 domain binding glutamate rich protein like 2 | 24.29 | 0.00 |
| 6q14.1 | ELOVL4 | Elongation of very long chain fatty acids 4 | 28.93 | 0.74 |
| 6q14.1 | TTK | TTK protein kinase | 14.89 | 0.01 |
| 6q14.1 | BCKDHB | Branched chain keto acid dehydrogenase E1 subunit beta | 21.82 | 0.00 |
| 6q14.1 | FAM46A | Family with sequence similarity 46 member A | 14.82 | 0.35 |
| 6q14.1 | **IBTK** | Inhibitor of Bruton tyrosine kinase | 26.49 | 0.90 |
| 6q14.1 | **DOPEY1** | DOPEY family member 1 | 25.26 | 1.00 |
| 6q14.1-q14.2 | PGM3 | Phosphoglucomutase 3 | 45.16 | 0.02 |
| 6q14.2 | ME1 | Malic enzyme 1 | 31.77 | 0.00 |
| 6q14.2 | **SNAP91** | Synaptosome associated protein 91 | 16.94 | 0.97 |
| 6q14.2 | CYB5R4 | Cytochrome b5 reductase 4 | 35.12 | 0.00 |
| 6q14.2 | MRAP2 | Melanocortin 2 receptor accessory protein 2 | 46.03 | 0.00 |
| 6q14.3 | **TBX18** | T-box 18 | 4.18 | 1.00 |
| 6q14.3 | NT5E | 5'-nucleotidase ecto | 11.56 | 0.00 |
| 6q14.3 | SNX14 | Sorting nexin 14 | 11.31 | 0.00 |
| 6q14.3 | **SYNCRIP** | Synaptotagmin binding cytoplasmic RNA interacting protein | 2.48 | 1.00 |
| 6q14.3 | HTR1E | 5-hydroxytryptamine receptor 1E | 49.02 | 0.02 |
| 6q14.3 | CGA | Glycoprotein hormones, alpha polypeptide | 22.43 | 0.25 |
| 6q14.3 | **ZNF292** | Zinc finger protein 292 | 26.55 | 1.00 |
| 6q14.3 | SMIM8 | Small integral membrane protein 8 | 29.48 | 0.04 |
| 6q15 | SLC35A1 | solute carrier family 35 member A1 | 23.12 | 0.21 |
| 6q15 | RARS2 | arginyl-tRNA synthetase 2, mitochondrial | 21.71 | 0.00 |
| 6q15 | ORC3 | origin recognition complex subunit 3 | 16.76 | 0.00 |
| 6q15 | **AKIRIN2** | akirin 2 | 17.80 | 0.98 |
| 6q15 | **CNR1** | cannabinoid receptor 1 | 1.58 | 0.16 |
| 6q15 | **RNGTT** | RNA guanylyltransferase and 5'-phosphatase | 2.89 | 0.76 |
| 6q15 | PNRC1 | proline rich nuclear receptor coactivator 1 | 23.15 | 0.84 |
| 6q15 | SRSF12 | serine and arginine rich splicing factor 12 | 27.22 | 0.02 |
| 6q15 | PM20D2 | peptidase M20 domain containing 2 | 45.56 | 0.00 |
| 6q15 | GABRR1 | gamma-aminobutyric acid type A receptor rho1 subunit | 31.63 | 0.00 |
| 6q15 | GABRR2 | gamma-aminobutyric acid type A receptor rho2 subunit | 29.38 | 0.00 |
| 6q15 | UBE2J1, UBC6E | ubiquitin conjugating enzyme E2 J1 | 29.26 | 0.86 |
| 6q15 | RRAGD, RAGD | Ras related GTP binding D | 24.06 | 0.81 |
| 6q15 | ANKRD6, KIAA0957 | ankyrin repeat domain 6 | 42.81 | 0.00 |
| 6q15 | LYRM2 | LYR motif containing 2 | 43.89 | 0.06 |
| 6q15 | **MDN1** | midasin AAA ATPase 1 | 38.89 | 1.00 |
| 6q15 | **BACH2** | BTB domain and CNC homolog 2 | 7.83 | 0.87 |
| 6q15 | **MAP3K7** | mitogen-activated protein kinase kinase kinase 7 | 2.75 | 1.00 |
| 6q16.1 | **EPHA7** | EPH receptor A7 | 2.76 | 0.99 |

All genes within 6q11-q15 with an haploinsufficiency score HI below 50%. The loss-of-function tolerance score pLI is also given. Genes printed in bold are highly likely to have a haploinsufficiency effect based on an HI below 10% or a pLI above 0.9.

The HI and pLI scores were derived from https://decipher.sanger.ac.uk and http://exac.broadinstitute.org, respectively, in September 2017.
